# Patriotism and the Nurse-Training Schools

**Published:** 1915-11-06

**Authors:** 


### PATRIOTISM & THE NURSE-TRAINING SCHOOLS.

### III. A GROUP OF NORTH-COUNTRY HOSPITALS.

The term "North Country" usually applied to the whole district above Birmingham is a wide one. From among the many famous institutions in Lancashire, Yorkshire, and Northumberland we append particulars of the nurses who have been sent from the following hospitals, all of which are fully recognising their national duty also in regard to the reception of sick and wounded soldiers in the wards.

### Bolton Infirmary.

The nurses now on active service are twelve in number; for some of them their nursing posts in the institution are being kept open, while others have resigned. T.F.N.S.: Nurses Dickens, Kent, and Portious-Scott. Q.A.I.M.N.S.R.: Nurses Magg, Richards, and Wilding at Alexandria, Nurse Claxton on a transport ship, Nurse Seely at Cairo, and Nurses Hutton, Dawson, Featherstone, and Sawyers on home service so far as is known.

### Hull Royal Infirmary.

The following is a list of nurses who have left the hospital since the war began, to join the Navy or Army Nursing Service: France.—Nurse M. Bannister, who had the honour of being mentioned by Sir John French in his dispatches for her good work among the wounded; Nurses Robinson, Hudson, Newton, Fewlass, Thompson, Holthouse, and Hadfield. Serbia.—Nurse Mehaffy. Egypt.—Nurse White. Hospital Ship.—Nurses Fowler and Boothman. Territorial Hospitals.—Nurses Ramsbotham, Lockwood, Best, Turner, Walker, Dearden, Kirkland, and Blanchard.

### Royal Southern Hospital, Liverpool.

The matron of this hospital, Miss L. E. Jolley, was a member of Q.A.I.M.N.S.R. before the war broke out, and left early in August 1914 to take up her duties abroad. For many months she worked on an ambulance train in France, and she is now attached to one of the casualty clearing stations out there. Sisters W. Hope, E. V. Monk, and M. Edwards, who belonged to the Territorial Nursing Force, were called out to the military hospital at Fazakerley, and the last-named has now been sent to France. Nurses E. Bowdler and M. Wilson joined the Liverpool Merchants' Mobile Hospital, and left England in March. Nurses M. Roberts, M. Silberbach, and L. Shaw are working at the Royal Naval Hospital, Plymouth. Nurse A. Buckley is on a hospital ship plying between England and the Dardanelles. Nurses G. Johnson, A. E. Casseeley, B. Culwick, G. Parry, and D. Teale, having joined Q.A.I.M.N.S.R., are all abroad. Nurse M. Clarke, also a member of the above service, has been in France since the beginning of the war, and to her has been awarded the Royal Red Cross. Three more nurses guaranteed for the Navy have not yet been called up. In this hospital fifty beds have been given to receive wounded soldiers, so the work of the nursing staff has greatly increased during the past year.

### Royal Infirmary, Liverpool.

In addition to members of the staff mentioned in our issue of Oct. 23, nine nurses were sent off a few days ago in response to a request from the War Office for nurses from the private staff for active service in the Mediterranean. Their names are: Nurses Bertwistle, Greenwood, Darlington, Clowes, Lea-Roberts, Taylor, Hime, Cartwright, Kellett.

### Royal Victoria Infirmary, Newcastle-on-Tyne.

Since September 1914 twenty V.A.D. members have been received in the wards for four weeks' experience, in successive relays, and the number trained under these conditions is now close on 300. In addition to this a limited number (from fourteen to twenty) of special probationers are receiving six months' training to fit them to serve as probationers in military hospitals. With this record of work accomplished within the institution, it is a striking fact that no fewer than twenty-six sisters and nurses have joined the Naval and Military Nursing Services since the outbreak of the war.

The assistant matron, Miss Williams, has been called up to take charge of the 2nd Eastern General Hospital, Hove. Q.A.R.N.N.S.R.: Sisters Jones, Smith, Grisdale; Nurses Robson, Purse, Fairweather, Boyes, Connor, Poole. Q.A.I.M.N.S.R.: Sisters McGregor, Main, Jackson, Nash; Nurses Williams, Winpenny, Goldsbrough, Lobley, Wilson, Redpath. T.F.N.S.: Sisters Williams, Hill, Preece, Gallant; Nurses Francis, Teasdale, and Bevan.

### Preston Royal Infirmary.

The assistant matron, Miss Charlesworth, a member of the T.F.N.S., is on war service, together with the following members of the staff: Sisters Ross and Stead; Nurses Hook, Whitmarsh, Pollett, and Robinson. These are serving in France. The number is augmented from time to time, for as the nurses finish their training they join the Q.A.I.M.N.S.R. or the T.F.N.S. A certain number of short-time probationers are being taken in for three or six months' work in preparation for service in military hospitals during the war.

### York County Hospital.

The following are the members of the nursing staff who have left for war service: Miss Kathleen Smith, T.F.N.S., was called up for duty in October 1914, and is at present night superintendent in the Territorial Hospital, Bristol. Miss Dorothy Beaton, eye-ward sister, left in February for Serbia, and has since become a member of Q.A.I.M.N.S.R. Nurse Ethel McGowan, Q.A.I.M.N.S.R., and Nurse S. Mathison, T.F.N.S., left in September. These are all who have gone on war service from the present staff, but many old York nurses are at the Front in various posts.

